# Policy Challenges in the Fight against Childhood Obesity: Low Adherence in San Diego Area Schools to the California Education Code Regulating Physical Education

**DOI:** 10.1155/2013/483017

**Published:** 2013-05-13

**Authors:** G. Consiglieri, L. Leon-Chi, R. S. Newfield

**Affiliations:** ^1^Division of Pediatric Endocrinology, Department of Pediatrics, Rady Children's Hospital San Diego, 3020 Children's Way MC 5103, San Diego, CA 92123, USA; ^2^Department of Pediatrics, University of California San Diego, 200 W Arbor Drive, San Diego, CA 92103, USA

## Abstract

*Objective*. Assess the adherence to the Physical Education (PE) requirements per California Education Code in San Diego area schools. *Methods*. Surveys were administered anonymously to children and adolescents capable of physical activity, visiting a specialty clinic at Rady Children's Hospital San Diego. The main questions asked were their gender, grade, PE classes per week, and time spent doing PE. *Results*. 324 surveys were filled, with 36 charter-school students not having to abide by state code excluded. We report on 288 students (59% females), mostly Hispanic (43%) or Caucasian (34%). In grades 1–6, 66.7% reported under the 200 min per 10 school days required by the PE code. Only 20.7% had daily PE. Average PE days/week was 2.6. In grades 7–12, 42.2% had reported under the 400 min per 10 school days required. Daily PE was noted in 47.8%. Average PE days/week was 3.4. Almost 17% had no PE, more so in the final two grades of high school (45.7%). *Conclusions*. There is low adherence to the California Physical Education mandate in the San Diego area, contributing to poor fitness and obesity. Lack of adequate PE is most evident in grades 1–6 and grades 11-12. Better resources, awareness, and enforcement are crucial.

## 1. Introduction

The World Health Organization declared obesity a global epidemic associated with negative health outcomes in 1997 [1]. In over three decades of national surveys in the USA, from 1971 until 2000, the prevalence of children and adolescents who are obese has tripled [2, 3]. Recently though, the prevalence has stabilized except for those with a BMI ≥97 percentile [4], such that in 6–19-year olds in the 2007-2008 survey more than 1 in 6 were obese (18.7% with BMI ≥ 95%), and over 1 in 3 were overweight or obese (34.7% with BMI ≥ 85%) [4]. One of the most effective ways of battling obesity is through legislation or policies that enable changes in lifestyle, whether it impacts diet or exercise. For such policies to be effective, their implementation ought to be monitored and enforced. Promotion of routine physical activity in children from preschool age on may help prevent the development of overweight and obesity and associated comorbidities, and the role of schools in achieving that has been recognized and emphasized in guidelines to help battle obesity [5, 6]. As not all children live in safe neighborhoods or are allowed to play outside, schools offer a safe place to accomplish physical activity, delivered with guidance. Schools have historically promoted physical activity and healthy eating, and since over the past 2 decades more than 99% of 7–13-year olds and over 95% of 14–17-year-old youths are enrolled in schools [7] and spend 5 days a week in school, the school environment can play an important role in preventing obesity [5, 6].

When we routinely asked overweight or obese children and adolescents that visited the pediatric endocrine clinic about the amount of PE they have, we were struck by how many have little or no PE time. Thus, we conducted a Physical Activity Survey given to all comers: children and adolescents from first through twelve grades who are able to participate in sports attending specialty clinics at Rady Children's Hospital San Diego (RCHSD). The survey assessed minutes of physical education per day and number of sessions per week in the school setting. This modest size, anonymous, self-reported survey supports prior data as in the 2007 California Health Interview Survey [8], which is not well recognized in the medical literature, showing inadequate PE time in California and should prompt stricter enforcement of the existing statutes. The current Physical Education requirement in California is as follows: Education Code Section 51210 that requires 200 minutes of physical education every ten school days for students in grades one through six. Education Code Section 51222 provides for 400 minutes of physical education every ten school days for students in grades seven through twelve. The requirement for physical activity does not include time spent during recess or lunch break. We describe in this paper the rate at which the Physical Education requirements of the state of California are met in San Diego County.

## 2. Methods

One-page surveys were handed to children and adolescents who maintained they were fit to practice PE. Surveys were filled together with their parent or guardian, while waiting to be seen for their clinic visit in a specialty clinic at RCHSD, located in southern California. The vast majority attended endocrinology and diabetes, or gastroenterology clinics, at that location. Patients reside mostly in San Diego County, with a small minority from adjacent Imperial and Riverside counties, representing both urban and rural communities. The samples are representative of San Diego County schools, as RCHSD is the only children's hospital in the metropolitan area of San Diego, serving all children in San Diego County. The surveys were anonymous, with no name, date of birth, or address requested. We did include their gender, ethnicity, their age in years, grade level, and whether they attended a charter school. They were asked how many times per week they have PE (not including physical activity at recess or lunch break, which is not included in the PE code either). This allowed us to arrive at the number of minutes per 10 days of school (double their 5-day week in school). We also asked about walking to/from school, and participation in a walking club at their school. The surveys were administered in two rounds, in Autumn 2011 and Spring 2012 semesters, to account for any seasonal variation. The sample size calculated to detect a difference in compliance rates with PE code between grades 1–6 and 7–12 with a *P* < 0.05, and a power of 80%, was 91 in each group. A lower sample size of 62 in each group was needed to detect a difference of 1 day in mean PE days per week among groups, assuming a rather wide SD of 2 days. We used a chi square analysis to compare the rates of participation among groups with the same PE requirements, and the rates of students participating in walking club or walking to/from school. We used the *t*-test to compare mean days of PE per week among groups.

## 3. Results

We collected 324 surveys that were completed properly, containing the key data elements. We present data only from 288 surveys, since 36 were from charter schools that are not mandated to comply with California PE code. However, PE participation rates in charter schools were very similar to the data we present in noncharter schools. Out of 288 surveys filled, 59% were females. Most were of Hispanic descent (43%), or Caucasian (34%), with smaller numbers of Asian (7%), or African American descent (6%), and 10% were of other or mixed ethnicity. There were 135 subjects from grades 1 through 6 and 153 from grades 7–12, that is, from middle school (64) and high school (89).

Time spent in PE per 10 days of school and weekly frequencies of PE are presented in Table 1. For grades 1–6, 90 (66.7%) had reported less than 200 min per 10 school days as required. None of those having PE only once weekly and only 2 out of 29 having PE twice weekly had ≥200 min of PE per 10 days, so, in general, having PE twice weekly is insufficient. Even with three times per week, 55% were under the 200 minutes mark. For grades 7–12, 65 (42.2%) had reported less than 400 min per 10 school days as required. A higher percentage of those in grades 1–6 did not meet the California PE mandate (*P* < 0.001), though the time required in grades 1–6 is lower. Although it appears that more adolescents in high school do not meet the required 400 min (47.2%) versus middle school (32.8%), this did not reach significance. Even if one would apply the 200-minute standard of lower grades, almost 1 in 4 (22.9%) students in grades 7–12 fails to receive that either, fewer in grades 7-8 (9.4%) versus in high school (30.3%). In contrast to grades 1–6, few in grades 7–12 had PE once or twice weekly (Table 1). Unlike in lower grades, all but one of the 12 who had PE twice weekly had less than 200 minutes of PE per 10 days, but only one met the 400-minute cutoff. Again, though the numbers are small, twice weekly PE fails to meet the required time for most students in middle and high schools. Even with three times per week, 54% were under the 400 minutes mark. Almost 17% had no PE, more so in high school and its final 2 grades; in grades 9-10, 7/54 (13%) had no PE, and in grades 11-12 as much as 16/35 (45.7%) had no PE. Daily PE was more common in grades 9-10 (46.3%) compared with grades 11-12 (34.3%), and a bit lower than in grades 7-8 (56.3%).

While we cannot compare the times spent in PE, as the mandate is different between grades 1–6 and grades 7–12, one can compare the frequency of PE per week (Table 1). More students in grades 7–12 had PE five times a week compared with grades 1–6 (*P* < 0.001), and less had PE once or twice weekly, respectively (*P* < 0.001); for no PE, the sample size was too small. Those results remain significant when examining those in grades 7-8 and 9–12 compared separately to those in Grades 1–6; an analysis was not performed on those with PE once a week or none due to the small numbers. As in Table 1, the average days of PE per week were lower in grades 1–6 versus grades 7–12 (*P* < 0.0001), due to the higher rate in grades 7-8, which was higher than in grades 9–12 (4.0 versus 3.0 days per week, *P* < 0.001); there was no statistical difference comparing PE days per week in grades 9–12 versus grades 1–6. There were no statistically significant differences in the number of PE days per week or the total time spent per 10 days in PE between females and males.

The rates of walking to school, from school, and to and/or from school are presented in Table 2. In grades 1–6, more children reported walking back from school rather than to school, and slightly more than a quarter had either walked to and/or from school. Most children who walked had done it both ways. In grades 7–12, the data shows more adolescents walking back from school, rather than to school. Walking rates to and/or from school was highest in grades 7-8 (40.6%), significantly higher than in grades 1–6 (*P* < 0.05), whereas the rates at high school were quite similar to those in grades 1 through 6. The numbers were too small to look at females versus males. Walking club participation was reported in 33.3% of children in grades 1–6, significantly higher (*P* < 0.01) than 15.6% in grades 7-8 and 13.5% of high school students.

## 4. Discussion

Based on the results of this moderate size study, it appears that there is currently low adherence to the PE requirements as stated in the California Education Code in San Diego schools. This is in agreement with prior reports in adolescents in California [8], and across the nation [9, 10], though there is wide variation among states [9]. The low adherence is more pronounced in this survey in grades 1–6, where only one in three children met the state requirement of at least 200 minutes per 10 days of school, whereas in grades 7–12, almost 58% met the guidelines of 400 minutes or more. Significantly fewer subjects in grades 1–6 had daily PE (5 times weekly) versus those in grades 7–12 (*P* < 0.001). Fewer high school students in grades 11-12 had daily PE (34.3%) than in grades 9-10 (46.3%), but a large number had no PE at all, 45.7% and 13%, respectively. To contrast, very few subjects in grades 7–12 had PE once weekly versus 33.3% of those from grades 1–6 in our survey (*P* < 0.001). Less than 10% of students who have PE twice a week meet the minimum requirements of PE, and only about a half of all students who have PE three times a week meet the state requirements. It is important to point out that the California PE requirements are lower than the National Association for Sports and Physical Education (NASPE), which recommends 150 minutes per week for elementary schools and 225 minutes for middle and high school (equivalent to 300 minutes and 450 minutes, resp., per 10 days of school standards in California). To meet the California PE code, we suggest that PE should be delivered 4-5 times per week or alternatively 3 times a week with longer periods than what is presently practiced. We did not ask about activity during recess or lunch break, as this is excluded from the PE code. We did inquire about other activities related to school that are meaningful in terms of total daily activity. Currently, due to our urban planning and parental concerns for their children's safety, less students walk to school. We found that for students in grades 1–6 and in grades 7–12, 26.7% and 33.3% either walked to and/or from school, respectively. This type of activity should be encouraged for all students. If more parents would walk with their children to/from elementary school, it would send a powerful signal to the children of the importance of physical activity while offering them safety and benefitting the parents as well. Participation in a walking club at school was reported in 33.3% of students in grades 1–6, which is significantly higher (*P* < 0.01) than the 15.6% in grades 7-8 and 13.5% in grades 9–12. This may be more appropriate for elementary schools due to peer perception. As mentioned previously, the data obtained from this study supports prior data that shows inadequate PE times [8–10]. This is particularly worrisome in the light of the increase of obesity rates since 1970 [2, 3]. In an observational study consisting of 814 third grade students in four states (including California), only 5.9% had PE five times per week [10]. Students had an average of 68.7 min/week in PE (i.e., 137.4 min per 10 day) and an average of 2.1 lessons per week [10]. Our study shows similar findings, with 6.5% of 3rd graders having had PE daily, with an average of 70.7 min/week, and 1.9 lessons per week. Additionally, a result of a nationwide study conducted in 2006 with 988 schools showed that only 3.8% of elementary schools, 7.9% of middle schools, and 2.1% of high schools provided PE daily [11]; this does not mean that all students in schools offering daily PE actually had PE daily. It is encouraging to see that the percent of students having daily PE in our survey is much higher than that (Table 1). However, there is still much more room for improvement, especially in grades 1–6, where the academic pressures should be less than grades 7–12, yet they have less PE on a daily basis. The average days/week of PE reported in this survey in San Diego area schools at grades 7–12 is 3.4 days/week, and it is higher than 2.7 days/week reported in a study from 2007, in 12–17-year olds in California [8]. In a national study from 2011 in high school students [9], only 52% of students attended PE per week. They reported no PE more often in 12th grade (62%) versus 9th grade (32%). The trend was similar in our cohort, but we are glad to report that the rates were lower, at 38.9% and 11.5%, respectively. The CDC also reported [9] higher daily PE in 9th grade (41%) versus 12th grade (24%). We found a similar trend comparing daily PE in grades 9-10 versus 11-12. This reflects the reality by which students usually do PE only for 2 years in high school to satisfy their academic credits but do not comply with the PE code. Nationally [9], the percentage of high school students who attended PE classes daily decreased from 42% in 1991 to 25% in 1995 and has recently remained stable at the level noted in 2011 (31%). The rates of daily PE reported by high school students in our survey (41.6%) are higher than the national rates, which is another piece of positive data in this cohort. According to the U.S. Surgeon General, regular physical activity is one of the most important ways to maintain and improve one's physical health, mental health, and overall wellbeing [12]. Physical activity scores of children in the 5th, 7th, and 9th grades that measured aerobic capacity, body composition, strength, and flexibility were strongly and positively correlated with language arts and mathematics proficiency [13]. It is unfortunate and short-sighted of schools that are increasing the time afforded to academics at the expense of physical activity classes. It goes against the extensive evidence that demonstrates better academic achievement in students who are more fit [13, 14].

Though there are some limitations to this study, they do not detract from the main message of low adherence to the PE code. We did not specifically ask if subjects had exemptions from PE due to religious or other causes, though we asked the surveys to be filled by students capable of doing PE. Nationally, the number of students' exempt from PE is relatively high, with 40% of elementary schools, 52% of middle schools, and 60% of high schools allowing an exemption from PE classes, particularly for students with permanent physical disabilities and those having religious reasons [15]. Since we used one-page surveys and gave them to randomly selected children and adolescents waiting for their clinical appointments, we did not ask in-depth questions about their physical education. We asked general questions used to analyze how much physical activity was done but did not track the types of activity and the intensity of activity among other factors. However, the data from this study and others offer important data that can benefit schools by giving guidance on how to best meet the California PE code.

Legislation for PE itself will not be sufficient. There needs to be proper enforcement of the legislation in order for students to meet the PE mandate and be more fit, if we want to have a healthier society with less of our youth becoming overweight and obese at an early age. It is the clinical impression of the author (RSN) that most parents are unaware of the California PE code or they assume that lunch and recess activity times actually count. Perhaps we can see a real change in adherence if the California PE code would require that parents should be notified in writing of the PE requirements or that schools that do not meet the mandate will get financially penalized. To make a meaningful change, PE should be mandated academically in high school in a way that is, compatible with the Education code that governs PE.

The results offered by this study demonstrate the need to address the issues of low adherence to physical education required in schools and to raise awareness of the importance of physical activity. The two biggest deficiencies in PE participation we found are in grades 1–6 and in grades 11-12. Pediatricians and other health care professionals should educate families on the PE code mandate, so parents can demand what is right for their children. If school administrators would know that the public is aware of the low adherence to the PE requirements and is concerned about the possible consequences to their children's weight and academic performance, it may prompt schools to increase PE time and offer better physical activity programs in schools. Schools are in a unique position to help decrease obesity prevalence in children and adolescents, and all measures must be taken to optimize that potential, beginning with enforcing current PE state mandates.

## Figures and Tables

**Table 1:** Rates of PE, and average time in PE.

	Grades 1–6 (*n* = 135)		Grades 7–12 (*n* = 153)		Grades 7-8 (*n* = 64)		Grades 9–12 (*n* = 89)	
<200 PE min/10 d	66.7%*							
<400 PE min/10 d			**42.2%**		**32.8%**		**47.2%**	

Rate and time in PE	%	Mean (SD)	%	Mean (SD)	%	Mean (SD)	%	Mean (SD)

No PE	1.5	0	16.9	0	4.7	0	25.8	0
PE × 1 per week	33.3*	77 (31)	2.6	100 (70)	1.5	60	3.4	120
PE × 2 per week	21.5*	136 (53)	7.8	265 (90)	7.8^+^	228 (114)	7.9^++^	291 (64)
PE × 3 per week	14.8	223 (89)	16.4	448 (208)	18.8	393 (196)	14.6	494 (214)
PE × 4 per week	8.2	335 (138)	8.5	458 (53)	10.9	480 (46)	6.7	433 (53)
PE × 5 per week	20.7*	343 (123)	47.8	533 (182)	56.3^§^	490 (130)	41.6^§^	575 (215)

PE days/week		2.57** (1.56)		3.4 (1.89)		3.98^§§,†^ (1.41)		2.98 (2.08)

Rates are in %, and average time is in minutes per 10 days (2 weeks of school). No SD presented if <3 observations. Comparing grades 1–6 to 7–12: *P < 0.001, **P < 0.0001. Comparing upper grades to grades 1–6: ^+^ *P* < 0.05, ^++^ *P* < 0.01, ^§^ *P* < 0.001, and ^§§^ *P* < 0.0001. Comparing grades 7-8 to 9–12: ^†^ *P* < 0.001.

**Table 2:** Rates of walking to or back from school.

Walking %	Grades 1–6	Grades 7–12	Grades 7-8	Grades 9–12
To school	18.5	19	28.1	12.4
From school	23.7	28.8	34.4	24.7
To and/or from	26.7	33.3	40.6*	28.1

Rates are in %. *P < 0.05 comparing grades 7-8 to grades 1–6.
